# POLD2 is activated by E2F1 to promote triple-negative breast cancer proliferation

**DOI:** 10.3389/fonc.2022.981329

**Published:** 2022-09-02

**Authors:** Zhen Zhang

**Affiliations:** Institute of Clinical Medicine, Jiangxi Provincial People’s Hospital, The First Affiliated Hospital of Nanchang Medical College, Nanchang, China

**Keywords:** TNBC, POLD2, E2F1, transcription factor, proliferation

## Abstract

Triple-negative breast cancer (TNBC) is a highly malignant breast cancer subtype with a poor prognosis. Improved insight into the molecular biology basis of TNBC progression is urgently needed. Herein, we reported that POLD2 was highly expressed in TNBC and patients with high POLD2 expression in their tumors had poor clinical outcomes. In functional studies, knockdown of POLD2 inhibited the proliferation of TNBC. Mechanistically, we revealed that transcription factor E2F1 directly bound to the promoter of POLD2 and regulated its expression in TNBC cells, which in turn contributed to the proliferation of TNBC. Additionally, rescue experiments validated that E2F1-mediated cell proliferation in TNBC was dependent on POLD2. Taken together, our results elucidated a novel mechanism of the E2F1-POLD2 axis in TNBC proliferation, and POLD2 may be a potential therapeutic target for TNBC treatment.

## Introduction

Breast cancer is the most prevalent cancer in women, among which triple-negative breast cancer (TNBC) accounts for approximately 15–20% (1). Compared with other subtypes of breast cancer, TNBC is associated with poorer overall survival and a higher probability of recurrence and death due to the absence of therapeutic targets. Thus, it is essential to reveal the molecular mechanisms involved in the carcinogenesis of TNBC to find new potential targets for therapy.

DNA polymerase delta (Polδ) is a major eukaryotic replicative polymerase, which is indispensable for efficient replication of the nuclear genome (2). Polδ consists of four subunits POLD1/p125, POLD2/p50, POLD3/p66 and POLD4/p12 in vertebrates (3). Among them, POLD1/p125 is the catalytic subunit while others are accessory subunits (4). In particular, Polδ has been suggested to play key roles in various physiological and pathological processes, including carcinogenesis. For example, POLD1 is overexpressed in hepatocellular carcinoma and breast cancer, which predicts poor prognosis in patients, suggesting that POLD1 may be a potential prognostic marker and promising therapeutic target in cancers (5, 6). Furthermore, research also indicated that common variations or germline mutations in POLD3 genes predispose to colorectal cancer (7, 8). And another research showed that POLD4 was low expressed in lung cancer patients, which was associated with poor prognosis (9). In addition, several studies have shown that POLD2 is aberrantly expressed in multiple cancers, including ovarian carcinoma (10) and glioblastoma (11). And interference of POLD2 with shRNA inhibited glioblastoma cell proliferation, cell cycle progression, invasiveness, and sensitized glioblastoma cells to chemo/radiation-induced cell death, indicating that POLD2 is a new potential therapeutic target for glioblastoma (11). However, the expression, clinical significance, and functions of POLD2 in TNBC are still unclear.

In the present study, we aimed to investigate the expression of POLD2 in TNBC and explore the potential regulatory role of POLD2 in the progression of TNBC. Furthermore, we will elucidate the molecular mechanisms by which endogenous POLD2 expression is regulated in TNBC cells.

## Materials and methods

### Database analysis

The TCGA BRCA cohort data were downloaded through the UCSC Xena platform (12). The expression profiles of POLD2 and its correlation with E2F1, SP1, and YY1 expression in TNBC were then analyzed. Kaplan-Meier and log-rank tests were used for the survival analysis. Transcription factors (TFs) implicated in the transcription of POLD2 were predicted using the UCSC ENCODE (13), JASPAR (14), and PROMO (15, 16) platform.

### Cell culture

MCF-10A, MDA-MB-231, and 293T cell lines were obtained from the American Type Culture Collection, and the SUM-159 cell line was obtained from Asterand Bioscience. Each cell line was authenticated by STR analysis. MCF-10A cells were cultured with DMEM/F12 supplemented with 5% horse serum, 100 U/mL penicillin, 100 μg/mL streptomycin, 20 ng/mL EGF, 0.5 μg/mL hydrocortisone, and 100 ng/mL cholera toxin. MDA-MB-231 cells were cultured with RPMI-1640 supplemented with 10% fetal bovine serum. SUM-159 and 293T cells were cultured with DMEM supplemented with 10% fetal bovine serum. All cells were cultured in a humidified incubator at 37°C with 5% CO_2_.

### Plasmids and transfections

Lentiviral systems for knockdown or expression of POLD2 and knockdown of E2F1 were performed as described previously (17). The primer sequences of POLD2 and E2F1 are listed in **Table S1**. Overexpression of E2F1 was performed using a pCMVHA-E2F1 vector (#24225, Addgene).

### RNA extraction and quantitative RT-PCR

Total RNA was extracted using TRIzol reagent (Invitrogen). RNA quantity and quality were determined by spectrophotometric measurements and agarose gel electrophoresis, respectively. Then, total RNA was reverse transcribed using Transcript First-Strand Synthesis Supermix (TransGen Biotech). qRT-PCR was conducted using the TransStart Green Q-PCR SuperMix kit (TransGen Biotech) on a CFX96 system (Biorad) and analysis was performed using the 2^-(ΔΔCT)^ method. GAPDH was used as a housekeeping gene. The primer sequences of GAPDH, POLD2, and E2F1 are listed in **Table S1**.

### Western blotting

Western blotting was performed as described previously (18). Briefly, we extracted total protein from the cells with RIPA lysis buffer and then determined its concentration by BCA Protein Assay (Thermo Scientific). Proteins were boiled in SDS loading buffer and analyzed by 10% SDS-PAGE. Subsequently, proteins were transferred to PVDF membranes and blocked by 5% skim milk. Then, membranes were immunoblotted against 1:1000 dilution of POLD2 antibody (Santa Cruz, sc-390583), 1:1000 dilution of E2F1 antibody (Proteintech, 66515-1-Ig), and 1:1000 dilution of β-actin antibody (Proteintech, 20536-1-AP). The membranes were then incubated with HRP-conjugated secondary antibodies, followed by exposure to an ECL detection reagent.

### CCK-8 assay

Cells (3×10^3^ per well) were plated in 96-well plates and cultured for 0, 24, 48, and 72 h at 37°C in 5% CO_2_. Then, 10 μl CCK-8 reagent (Dojindo) was added and incubated for 2 h at 37° C. Subsequently, absorbance at 450 nm was measured. And cell proliferation curves were assessed by two-way analysis of variance (ANOVA) after cell seeding 0, 24, 36, 48, 60, and 72 hours.

### Colony formation assay

Cells (1×10^3^ per well) were plated in 6-well plates and cultured for 2 weeks at 37°C in 5% CO_2_. Then, the colonies were fixed in methanol and stained with 1% crystal violet for quantification.

### EDU incorporation assays

EdU incorporation was performed using EdU Imaging Kits-Cy3 (APExBIO) according to the manufacturer’s protocols. Briefly, cells were incubated with 50 μM EdU for 2 h and then fixed in 4% paraformaldehyde for 15 min. Subsequently, cells were permeabilized with 0.5% Triton X-100 for 20 min, and stained with a click reaction cocktail for 30 min. Cell nuclei were stained with 5 μg/mL Hoechst 33342 for 5 min. The number of EdU-positive cells was counted and photographed under a microscope.

### Luciferase assay

The human WT-POLD2 and MUT-POLD2 promoters were cloned into the pGL3−basic vector (Promega). Primer sequences are provided in **Table S1**. The pGL3−basic, pGL3-POLD2-WT, or pGL3-POLD2-MUT promoter vector and E2F1 expression vector were co-transfected into cells *via* Lipofectamine 2000 reagent (Invitrogen). After 48 h, the luciferase activities were measured using the dual-luciferase reporter assay system (Promega) according to the manufacturer’s protocols.

### ChIP-qPCR

ChIP-qPCR was performed as described previously (19), using an anti-E2F1 antibody (Proteintech, 66515-1-Ig). And the specific primers are listed in **Table S1**.

### Statistical analysis

Statistical analysis was performed using GraphPad Prism software. All the data are presented as the mean ± standard deviation. For correlation analyses of gene expression in tissue samples, the spearman correlation test was used. The survival of TNBC patients was analyzed with the Kaplan-Meier and log-rank tests. The statistical analysis involving two groups was performed by Student’s t-test. A *P* value less than 0.05 was considered significant.

## Results

### POLD2 is highly expressed in TNBC and correlated with poor prognosis of TNBC patients

**Figure 1:**
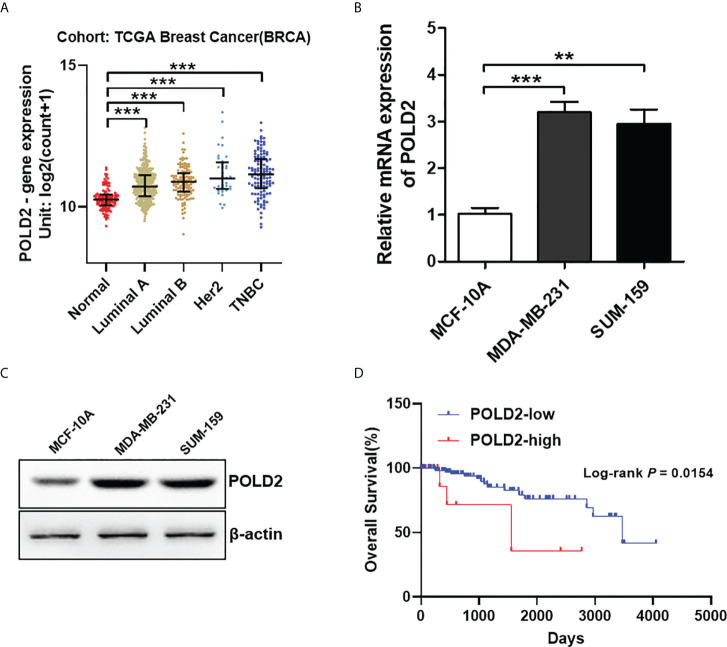
POLD2 is highly expressed in TNBC and correlated with the poor prognosis of TNBC patients. **(A)** The expression of POLD2 in different subtypes of breast cancer (i.e., TNBC, Luminal A, Luminal B, and Her2 positive) tissues and adjacent normal tissues from the TCGA database. ***P < 0.001. **(B, C)** The mRNA and protein expression of POLD2 in normal breast epithelial cell line MCF-10A and TNBC cell lines MDA-MB-231 and SUM-159. ***P < 0.001, **P < 0.01. **(D)** Kaplan–Meier curves show that patients with high expression of POLD2 in their TNBC tumors have shorter overall survival than patients with low expression from the TCGA database. *P* = 0.0154 by log-rank test. Data represent the mean ± SD of three independent experiments.

To explore the function of POLD2 in TNBC, we first investigated the expression of POLD2 in normal breast and different subtypes of breast cancer (i.e., TNBC, Luminal A, Luminal B, and Her2 positive) from The Cancer Genome Atlas (TCGA) database. As shown in **Figure 1A**, TNBC, Luminal A, Luminal B, and Her2 positive breast cancers all maintain high levels of POLD2 expression compared to normal breast tissue samples. However, it should be noted that POLD2 displayed the highest expression in TNBC compared with the other subtypes of breast cancer. Thus, we then examined the expression of POLD2 in TNBC and normal breast epithelial cell lines. The results showed that POLD2 mRNA and protein levels in TNBC cell lines including MDA-MB-231 and SUM-159 were higher than that in normal cell line MCF-10A (**Figures 1B, C** **)**. Additionally, survival analysis revealed that high POLD2 expression was related to poor overall survival in TNBC patients, as shown in **Figure 1D**, patients with high POLD2 expression had a shorter median overall survival (1556 days) than the low POLD2 expression (3472 days). Taken together, these results suggest that upregulated POLD2 is associated with TNBC progression.

### Inhibition of POLD2 reduces TNBC proliferation

**Figure 2:**
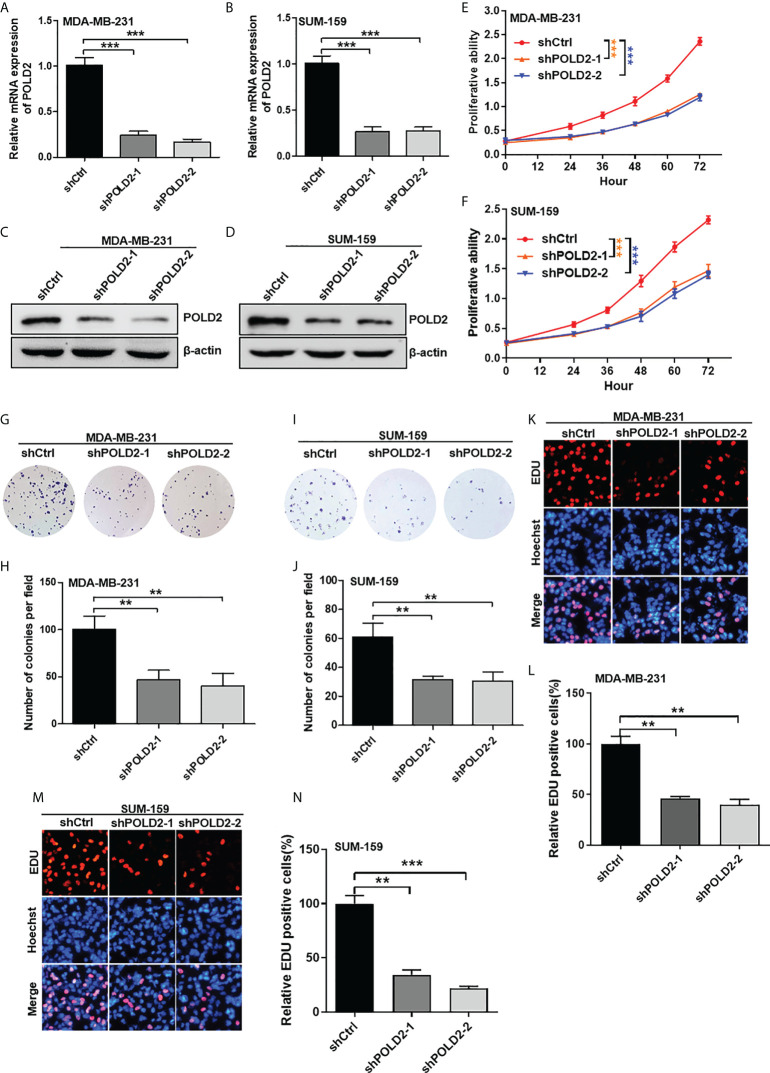
Inhibition of POLD2 reduces TNBC proliferation. **(A–D)** qRT-PCR **(A, B)** and Western blot **(C, D)** analysis of POLD2 knockdown in MDA-MB-231 and SUM-159 cells. ***P < 0.001. **(E, F)** The effect of POLD2 on MDA-MB-231 and SUM-159 cell viability was determined by a CCK-8 assay. ***P < 0.001. **(G–J)** The effect of POLD2 on MDA-MB-231 and SUM-159 cell colony formation abilities was determined by colony formation assay. **P < 0.01. **(K–N)** The effect of POLD2 on MDA-MB-231 and SUM-159 cell proliferation abilities was determined by EDU incorporation assay. ***P < 0.001, **P < 0.01. Data represent the mean ± SD of three independent experiments.

To investigate the roles of POLD2 in the malignant progression of TNBC, we firstly downregulated POLD2 expression by using shRNAs in MDA-MB-231 and SUM-159 cells **(** **Figures 2A–D** **)**. Then, the CCK-8 assay revealed that inhibition of POLD2 reduced cell viability **(** **Figures 2E, F** **)**. Downregulation of POLD2 also inhibited colony formation in TNBC cells **(** **Figures 2G–J** **)**. *Via* EDU incorporation assay, proliferation abilities of cells were demonstrated to be significantly attenuated after POLD2 inhibition **(** **Figures 2K–N** **)**. Collectively, these results demonstrate that POLD2 is essential for TNBC proliferation.

### E2F1 transcriptionally regulates POLD2 expression in TNBC cells

**Figure 3:**
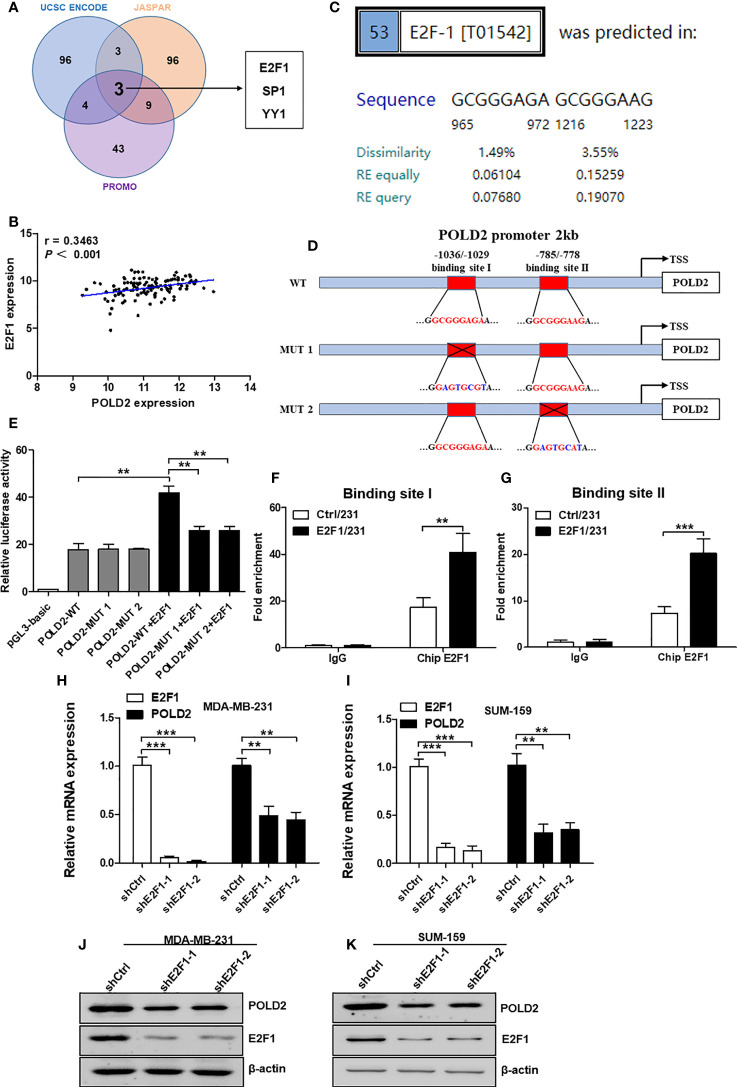
E2F1 transcriptionally regulates POLD2 expression in TNBC cells. **(A)** Three websites, UCSC ENCODE, JASPAR, and PROMO, were employed to predict the potential TFs. Three TFs were possible candidates. **(B)** A correlation between POLD2 expression and E2F1 expression in TNBC samples was analyzed by TCGA databases. **(C)** The binding sites of E2F1 within the promoter region of the POLD2 gene (~2 kb) were predicted by using the PROMO platform. **(D)** The diagram showed the putative E2F1 binding sites (EBSs) located in the promoter region of the POLD2 gene. Luciferase reporter vectors containing WT-POLD2-promoter or MUT-POLD2-promoter were constructed. **(E)** Luciferase reporter assays showed that POLD2 promoter enhanced luciferase activities in E2F1-expressing MDA-MB-231 cells, whereas those carrying mutant sites showed strong repression of luciferase activities. **P < 0.01. **(F, G)** Overexpression of E2F1 significantly enhanced its recruitment to the endogenous POLD2 promoter as confirmed by a ChIP-qPCR assay. ***P < 0.001, **P < 0.01. **(H–K)** The mRNA **(H, I)** and protein **(J, K)** expression of E2F1 and POLD2 in shCtrl/231, shE2F1/231, shCtrl/159 and shE2F1/159cells. ***P < 0.001, **P < 0.01. Data represent the mean ± SD of three independent experiments.

To determine the regulatory mechanism of POLD2 expression in TNBC, we predicted the transcription factors (TFs) implicated in the transcription of POLD2 using the UCSC ENCODE, JASPAR, and PROMO public prediction websites. And three TFs were screened out, including E2F1, YY1, and SP1 (**Figure 3A**). Of note, the expression levels of E2F1 showed a highly positive correlation with POLD2 in TNBC samples (**Figure 3B** and **Figures S1A, B**), indicating that E2F1 might regulate POLD2 expression. As a member of the E2 promoter binding factor (E2F) family, E2F1 regulates gene transcription by binding to the target genes promoter through the DNA binding domain (DBD) (20). Thus, we predicted the potential binding sites for E2F1 within the promoter region of the POLD2 gene (~2 kb) using the PROMO platform. And results showed that there are two major E2F1 binding sites (EBSs) that exist in the POLD2 promoter region (**Figure 3C**). Then, to validate the E2F1 transcriptionally regulates the expression of POLD2 *via* predicted EBSs, a dual-luciferase reporter assay was conducted to detect the luciferase activity of wild type POLD2 promoter (WT-POLD2 promoter) and mutant POLD2 promoter (MUT-POLD2 promoter) that respectively co-transfected with control or E2F1 overexpression vector in MDA-MB-231 cells **(** **Figure 3D** **)**. The results showed that overexpression of E2F1 could upregulate the luciferase activity of WT-POLD2-promoter, while mutated EBSs resulted in diminished luciferase activity **(** **Figure 3E** **)**. Of note, Chip-qPCR results showed that overexpression of E2F1 significantly increased its recruitment to the EBSs in the POLD2 promoter **(** **Figures 3F, G** **)**, demonstrating a predominant role for EBSs in the activation of POLD2 transcription by E2F1. In addition, the results of qRT-PCR and western blot showed that E2F1 knockdown significantly reduced the mRNA and protein expression of POLD2 in MDA-MB-231 and SUM-159 cells **(** **Figures 3H–K** **)**. Altogether, these results indicated that E2F1 activates POLD2 transcription and induces POLD2 expression in TNBC.

### POLD2 antagonizes the effect of E2F1 knockdown in regulating TNBC proliferation

**Figure 4:**
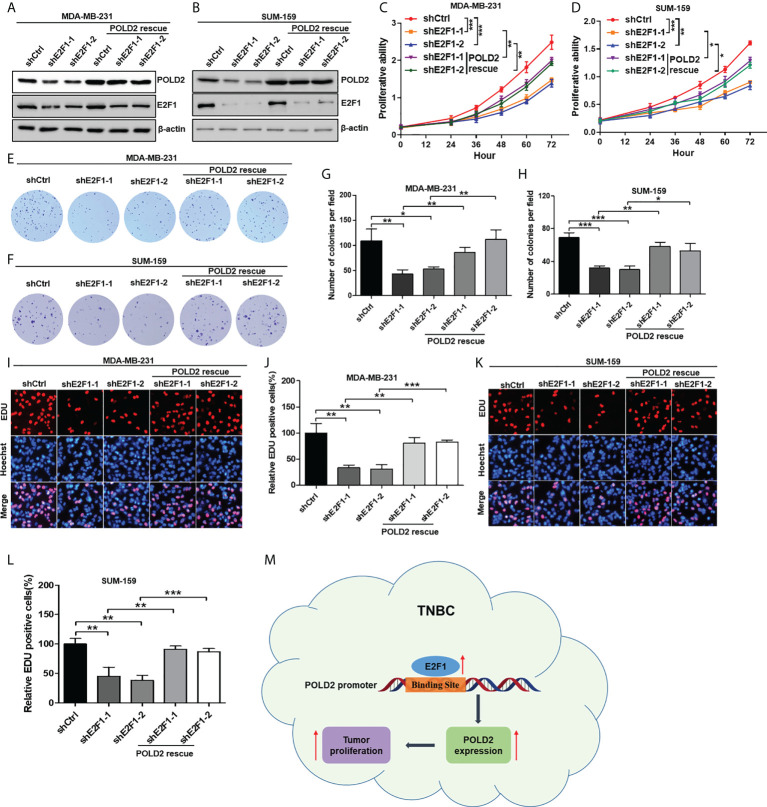
POLD2 antagonizes the effect of E2F1 knockdown in regulating TNBC proliferation. **(A, B)** The protein expression of E2F1 and POLD2 in shCtrl/231, shE2F1/231, shCtrl/159, and shE2F1/159 cells in the presence or absence of overexpressed POLD2. **(C, D)** The effect of E2F1 on MDA-MB-231 and SUM-159 cells viability in the presence or absence of overexpressed POLD2 was determined by CCK-8 assay. ***P < 0.001, **P < 0.01, *P < 0.05. **(E–H)** The effect of E2F1 on MDA-MB-231 and SUM-159 cells’ colony formation abilities in the presence or absence of overexpressed POLD2 was determined by colony formation assay. ***P < 0.001, **P <0.01, *P < 0.05. **(I–L)** The effect of E2F1 on MDA-MB-231 and SUM-159 cells’ proliferation abilities in the presence or absence of overexpressed POLD2 was determined by EDU incorporation assay. ***P < 0.001, **P <0.01, *P < 0.05. **(M)** A working model of the E2F1-POLD2 signaling pathway in TNBC proliferation. Data represent the mean ± SD of three independent experiments.

To further determine whether E2F1 could mediate TNBC proliferation *via* regulating POLD2 expression, POLD2 was overexpressed on the basis of E2F1 knockdown in MDA-MB-231 and SUM-159 cells **(** **Figures 4A, B** **)**. Then CCK-8 assay revealed that inhibition of E2F1 reduced cell viability; however, this effect was significantly weakened by the rescue of POLD2 expression **(** **Figures 4C, D** **)**. Downregulation of E2F1 also inhibited colony formation in TNBC cells, while overexpression of POLD2 attenuated this inhibitory effect **(** **Figures 4E–H** **)**. *Via* EDU incorporation assay, proliferation abilities of TNBC cells were demonstrated to be significantly attenuated after E2F1 inhibition, while rescued by POLD2 expression **(** **Figures 4I–L** **)**. Thus, E2F1 could promote cancer proliferation *via* upregulating POLD2 expression in TNBC, indicating that the E2F1-POLD2 signaling pathway is a driver of TNBC proliferation and could be a potential therapeutic target for the treatment of TNBC.

## Discussion

POLD2, also referred to as DNA Polymerase Delta Subunit P50, is a subunit of the DNA polymerase delta complex, which serves as a scaffold for the proper formation of Polδ by interacting with POLD1/p125, POLD3/p66, and POLD4/p12 (21). The human POLD2 gene was initially cloned from a panel of human-hamster hybrid cell lines, and located on human chromosome 7p13 (22). POLD2 has been shown to be essential for maintaining normal mammalian cellular systems function through regulating purine metabolism, DNA replication, nucleotide excision repair, mismatch repair, and base excision repair pyrimidine (23–25). It has been reported in a previous study that biallelic mutations in POLD2 lead to reduced functionality of the polymerase delta complex as the underlying cause of an autosomal-recessive syndromic immunodeficiency (25). Notably, emerging evidence from clinical and experimental studies has shown that dysregulated POLD2 expression occurs in a variety of tumors. For example, POLD2 was highly expressed in ovarian carcinomas, and patients with high POLD2 expression had a tendency of shorter survival, suggesting that POLD2 was a predictor of the prognosis for ovarian cancer patients (10, 26). Likewise, another study found that high expression of POLD2 was associated with cisplatin-based therapy resistance in bladder urothelial carcinoma, which may contribute to the poor prognosis of patients (27). A recent study also showed that POLD2 expression was significantly elevated in glioma tissues compared with non-tumor tissues, and high expression of POLD2 was associated with shorter survival in glioma patients (11). Consistent with these studies, we show that POLD2 expression is significantly higher in TNBC compared with the normal breast and patients with high POLD2 expression in their tumors have poor clinical outcomes, indicating that POLD2 is a prognostic indicator in patients with TNBC. Moreover, our researches show that inhibition of POLD2 expression significantly reduces TNBC proliferation, which is consistent with the findings of Q. Xu, et al. showing that shRNA-based POLD2 expression knockdown inhibited glioblastoma development (11), indicating that developing POLD2 inhibitors may provide a potential therapeutic strategy to inhibit the progression of malignancies in future research.

POLD2 expression is upregulated in various cancers, but little is known about how the expression of POLD2 is regulated. Using microarray, expression of POLD2 was found to be down-regulated by the exogenous overexpression of PTEN tumor suppressor (28). However, it is unclear whether PTEN regulated the expression of POLD2 directly or indirectly. And another study in glioblastoma stem-like cells showed that SOX2 significantly induced the expression of POLD2 by binding to the POLD2 promoter (11). In the present study, we demonstrate for the first time that POLD2 expression is upregulated directly by the E2F1 transcription factor in TNBC, which expands the understanding of the regulatory mechanisms of POLD2 expression. However, more investigations are needed to elucidate the other potential regulators of POLD2 expression, such as post-transcriptional modification, post-translational modification, genetic variability or methylation of the POLD2 gene.

Furthermore, our findings established that the E2F1-POLD2 signaling pathway contributes to TNBC proliferation. Considering that E2F1 dysregulation is a common feature among different tumor types and can be a major cause of cell proliferation (29), our research may provide a new insight into that E2F1 induces cell proliferation partially through activating POLD2 transcription, which expanded on the mechanisms of E2F1 promotes cell proliferation. It should be noted that our study has demonstrated high expression of POLD2 was caused by E2F1 in TNBC proliferation, but how exactly POLD2 promotes TNBC proliferation remains unclear. Therefore, more studies need to be carried out to investigate the potential mechanisms of POLD2 regulating the proliferation of TNBC. And besides its role in TNBC proliferation, other functions of POLD2 in TNBC progression remain unknown. For example, whether POLD2 promotes TNBC invasion, metastasis, radioresistance or chemoresistance warrants future investigation.

## Conclusions

In this study, we demonstrated that POLD2 was overexpressed in TNBC and high expression of POLD2 predicted the poor clinical outcome of TNBC patients. And inhibition of POLD2 significantly reduced TNBC proliferation, indicating that POLD2 functioned as an oncogene in TNBC. At the molecular level, we identified that transcription factor E2F1 was directly bound to the promoter of POLD2 and regulated its expression, which consequently conferred TNBC proliferation. Collectively, our findings revealed that the E2F1-POLD2 axis played a key role in the proliferation of TNBC, which could be utilized as a novel therapeutic target for TNBC treatment **(** **Figure 4M** **)**.

## Data availability statement

The original contributions presented in the study are included in the article/**Supplementary Material**. Further inquiries can be directed to the corresponding author.

## Author contributions

ZZ conceived and designed the study, developed methodology, analyzed and interpreted the data (for example, statistical analysis, biostatistics, computational analysis) and wrote the paper. The author confirms being the sole contributor of this work and has approved it for publication.

## Funding

This work is supported by grants from the Science and Technology Research Project of Jiangxi Provincial Department of Education (No. GJJ218906).

## Conflict of interest

The authors declares that the research was conducted in the absence of any commercial or financial relationships that could be construed as a potential conflict of interest.

## Publisher’s note

All claims expressed in this article are solely those of the authors and do not necessarily represent those of their affiliated organizations, or those of the publisher, the editors and the reviewers. Any product that may be evaluated in this article, or claim that may be made by its manufacturer, is not guaranteed or endorsed by the publisher.
