# Supplementary material for: POLD2 is activated by E2F1 to promote triple-negative breast cancer proliferation
Source: Front Oncol. 2022 Sep 2;12:981329. doi: 10.3389/fonc.2022.981329 (PMC9479206; doi:10.3389/fonc.2022.981329)
Supplement: Supplementary file 1 [file DataSheet_1.pdf]

Supplementary Information

This file includes: Fig. S1 and Tables S1.

Fig. S1.

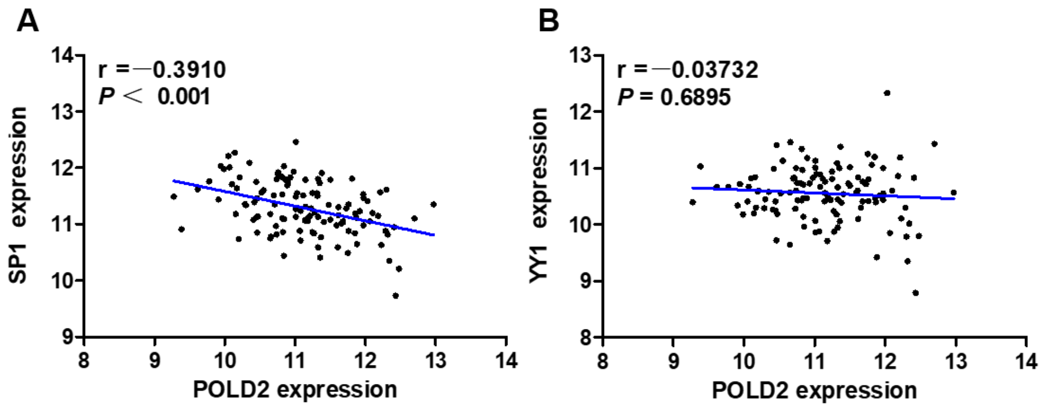

Supplementary Figure 1. (A) A correlation between POLD2 expression and SP1 expression in TNBC samples was analyzed by TCGA databases. **(B)** A correlation between POLD2 expression and YY1 expression in TNBC samples was analyzed by TCGA databases.

Table S1. Primers used in this study.

**Construction of POLD2 expression vector**	
POLD2 forward	5'-ATGTTTTCTGAGCAGGCTGCCC-3'
POLD2 reverse	5'-TCAGGGGCCCAGCCCCAGGCCT-3'
**Construction of POLD2 promoter vector**	
WT-POLD2 promoter forward	5'-AAAGTATTTGACAAAATTCAACACT-3'
WT-POLD2 promoter reverse	5'-GCTAATCCCCGCGCGGCTT-3'
MUT1-POLD2 promoter forward	5'-CTGAGGAGTGCGTATCACTGGAGCCTGG-3'
MUT1-POLD2 promoter reverse	5'-AGTGATACGCACTCCTCAGCCTCCCGA-3'
MUT2-POLD2 promoter forward	5'-GCGCGGAGTGCATAGCCCGGGAAGCTTA -3'
MUT2-POLD2 promoter reverse	5'-CGGGCTATGCACTCCGCGCGCTGATTCT -3'
**Construction of shRNA vector**	
shPOLD2-1	5'-GGCAGTATGCCCACATTTA-3'
shPOLD2-2	5'-GGTGGAGGACTATTGCTTTG-3'
shE2F1-1	5'-GCTATGAGACCTCACTGAA-3'
shE2F1-2	5'-GGACTCTTCGGAGAACTTTC-3'
**Quantitative RT-PCR**	
POLD2 forward	5'-CCACCCGCCTCATCCAAAT-3'
POLD2 reverse	5'-CCAAGACCAGCTCGTCATCTG-3'
E2F1 forward	5'-ACGCTATGAGACCTCACTGAA-3
E2F1 reverse	5'-TCCTGGGTCAACCCCTCAAG-3
GAPDH forward	5'-GGAGCGAGATCCCTCCAAAAT-3'
GAPDH reverse	5'-GGCTGTTGTCATACTTCTCATGG-3'
**Chip-qPCR**	
Binding Site I forward	5'-CTTAAAGTAGCCAGGCGTGGTG-3'
Binding Site I reverse	5'-AGGCAGCGTCTTGCTCTGTG-3'
Binding Site II forward	5'-AGGGAAGTAGCAGAACAGTAGCG-3'
Binding Site II reverse	5'-AGTGCTGTCTCCCACTTTGG-3'
